# Age-Dependent Honey Bee Appetite Regulation Is Mediated by Trehalose and Octopamine Baseline Levels

**DOI:** 10.3390/insects12100863

**Published:** 2021-09-24

**Authors:** İrem Akülkü, Saleh Ghanem, Elif Filiztekin, Guntima Suwannapong, Christopher Mayack

**Affiliations:** 1 Molecular Biology, Genetics and Bioengineering, Faculty of Engineering and Natural Sciences, Sabancı University, 34956 İstanbul, Turkey; iremakulku@sabanciuniv.edu (İ.A.); salehbader@sabanciuniv.edu (S.G.); efiliztekin@sabanciuniv.edu (E.F.); christopher.mayack@sabanciuniv.edu (C.M.); 2 Biological Science Program, Faculty of Science, Burapha University, Chon Buri 20131, Thailand

**Keywords:** aging, *Apis mellifera*, appetite regulation, biogenic amines, energetic homeostasis, sugar levels

## Abstract

**Simple Summary:**

Appetite regulation is an important function necessary to maintain energetic balance, but how honey bees accomplish this could vary as they age because they go through a number of behavioral and physiological changes during development. Here, we determine if the amount of trehalose, which is a sugar found in the hemolymph of honey bees, influences appetite levels and if this interacts with the octopamine neurotransmitter in the bee brain. To accomplish this, we decreased trehalose levels in the hemolymph by injecting an inhibitor of trehalose synthesis. In addition, we increased octopamine levels in the brain by injecting it with a syringe. We found that octopamine and trehalose interact to increase appetite in the two older age classes of bees, beyond just treating the bees with octopamine. The youngest age class did not respond to either treatment. Our results suggest that older honey bees may have an alternative pathway for regulating appetite that uses sugar levels in their hemolymph to communicate to the brain how hungry they are and that octopamine is responsible for elevating appetite levels when the bee is hungry. This pathway is different from how vertebrates regulate their appetite levels based on glucose levels in the blood.

**Abstract:**

There are multiple feedback mechanisms involved in appetite regulation, which is an integral part of maintaining energetic homeostasis. Older forager honey bees, in comparison to newly emerged bees and nurse bees, are known to have highly fluctuating hemolymph trehalose levels, higher appetite changes due to starvation, and higher octopamine levels in the brain. What remains unknown is if the hemolymph trehalose and octopamine levels interact with one another and how this varies as the bee ages. We manipulated trehalose and octopamine levels across age using physiological injections and found that nurse and forager bees increase their appetite levels due to increased octopamine levels in the brain. This is further enhanced by lower trehalose levels in the hemolymph. Moreover, nurse bees with high octopamine levels in the brain and low trehalose levels had the same appetite levels as untreated forager bees. Our findings suggest that the naturally higher levels of octopamine as the bee ages may result in higher sensitivity to fluctuating trehalose levels in the hemolymph that results in a more direct way of assessing the energetic state of the individual. Consequently, forager bees have a mechanism for more precise regulation of appetite in comparison to newly emerged and nurse bees.

## 1. Introduction

Appetite regulation plays a central role in maintaining energetic homeostasis [1,2]. In comparison to vertebrates, insects, due to their small size, have limited fat storage and instead rely upon the buildup of nutrients circulating around the hemolymph as an energy reserve [3,4]. Honey bees (*Apis mellifera*) only have three sugars found in their hemolymph: glucose, fructose, and trehalose. Only trehalose is known to dynamically fluctuate, possibly reflecting the energetic state of the individual [5,6,7,8]. Therefore, it has been proposed that the hemolymph trehalose levels may act as an indicator of the hunger state for the honey bee and other insects that use it as an energy storage mechanism [9,10]. This is despite the fact that the cells that make up the insect are still using glucose as their source of energy. Supporting this notion, when glucose and fructose levels are abundant, hemolymph trehalose is synthesized. When there is a depletion of glucose, trehalose is broken down into glucose to sustain life activities such as foraging for pollen and nectar [8,11]. Trehalose also aids in the regulation of foraging on an individual level, crop emptying rates, and foraging decisions [9,12,13].

What is less known is if trehalose levels are connected to appetite regulation independent of hemolymph glucose levels and, if so, how are the two regulatory systems interfaced with one another. A likely candidate for the connection between hemolymph trehalose levels and appetite regulation is the biogenic amine octopamine. Octopamine is equivalent to adrenalin and noradrenalin in vertebrates and is known to be released during times of stress and starvation [14]. Octopamine is highly conserved among invertebrates and serves as a hormone, neurotransmitter, and neurohormone, with pleiotropic effects [15]. An increase in octopamine levels is associated with increased appetitive associative learning, metabolism, activity, waggle dancing behavior, foraging, accelerated age polyethism, and is known to increase glucose levels from the mobilization of glycogen and fat stores [16,17,18,19,20,21,22,23,24]. Previous studies have shown that it can, in fact, increase gustatory sensitivity to sucrose and appetite levels in honey bees [25].

The honey bee undergoes consistent and predictable behavioral and physiological changes as it ages; this is known as age polyethism [26]. For instance, newly emerged bees are considered to be adult bees that have spent less than 24 h outside of the honey comb cell. These bees immediately start cleaning the honey comb where they emerged from and some of them will eventually attend the queen. Eventually these bees will become nurse bees around 5–7 days after hatching, which is associated with performing nest maintenance activities. At around 3–4 weeks of age, the honey bee goes from spending the majority of time inside the hive, to flying around outside the hive as a forager to collect nectar and pollen [27,28]. Not surprisingly, as the bee ages and transitions from one behavioral task to the next, there is also an increase in metabolism and appetite levels as well [29,30,31,32]. As the bee ages, there is also a corresponding increase in octopamine, dopamine, and serotonin levels, but a lowering in tyramine levels in the brain [33,34]. When transitioning from an in-hive to a forager bee, there is a reduction in fat stores to increase flight efficiency [35], an increase in vitellogenin (Vg) [36] and juvenile hormone (JH) [37,38], and an increased reliance upon trehalose sugar levels to fuel flight when foraging [8]. Previously, it has been shown that under fed and starved conditions the forager bee’s trehalose levels fluctuates the most in comparison to the newly emerged and nurse bees and this corresponds to a significant increase in octopamine levels in the brain [33]. Therefore, we suspect that forager bees may have different appetite regulatory mechanisms that are more in tune with hemolymph sugar levels in comparison to the newly emerged and nurse bees that have more glycogen and fat stores [39]. In addition, we hypothesize that high baseline octopamine levels, which are naturally relatively high in forager bees, in comparison to newly emerged and nurse bees, is responsible for not only higher appetite levels in this age class, but also interacts with the trehalose levels in the hemolymph as a way to regulate appetite. As evidence of this, we predict that nurse bees with elevated levels of octopamine will have similar appetite levels as forager bees and will also be more sensitive to changes in hemolymph trehalose levels in relation to appetite regulation.

Here, we inject newly emerged, nurse, and forager bees with octopamine and fumarate (Tyramine β-hydroxylase inhibitor) to increase and decrease octopamine levels in the bee brain, respectively [40]. We then inject a 10% sorbose solution into the thorax of the bee as this is known to lower trehalose levels [41]. Afterwards, we measure the bee’s appetite using the proboscis extension response (PER) assay [42] to determine if octopamine interacts with trehalose levels to increase appetite levels and to document how this changes during the aging process of the bee.

## 2. Materials and Methods

### 2.1. Honey Bee Collection of Known Ages

We collected brood frames from 5 source colonies which were then brought back to the laboratory to be placed in an incubator set to 32 °C with a relative humidity (RH) of around 70%. The newly emerged bee age class was comprised of bees that were removed within 24 h of hatching from the brood frame and used immediately for experimentation, while the other bees were released back into a source colony to be collected 1 and 4 weeks later, as nurse and forager bees, respectively. The hatched bees were randomly mixed to account for differences in genetic background. After hatching, all three age classes were marked with a dot of enamel paint (Testors, Vernon Hills, IL, USA) on the back of their thorax for recapturing. All bees were individually collected using a 20-mL glass vial and were chilled on ice until immobilization. These bees were harnessed in a plastic drinking straw using 1-mm-wide duct tape strips so that their proboscis could freely extend.

### 2.2. Appetite Regulation Experiment

**Figure 1:**
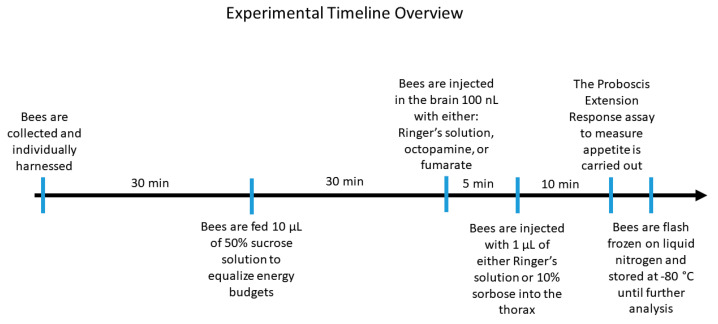
On overall timeline of the experimental procedure conducted with harnessed honey bees. The 10- and 5-min injection times were chosen based on when the maximum effect of the brain and thorax injections would be expected in relation to when the appetite was measured using the Proboscis Extension Response assay. The bees were fed with 10 µL of 50% sucrose solution to equalize their energy budgets at the start of the experiment.

A total of 50 bees were harnessed at one time per treatment. Bees that did not recover from the chill coma were discarded. This was typically less than 5% of the harnessed bees. The order of treatments was randomized to account for seasonal effects. The bees were given 30 min to acclimate to the harness and then were fed 10 µL of 50% sucrose solution to equalize their energy budgets at the start of the experiment [13]. Following the methods of [43], the medial ocelli lens was removed using a microscalpel (Feather, Osaka, Japan) and 100 nL of brain injections were carried out through the ocellar tract using a 30-gauge (BD Precision Glide, Franklin Lakes, NJ, USA) needle and a 10-µL Hamilton Syringe (Reno, NV, USA), placed on a micromanipulator (World Precision Instruments, Friedberg, Germany). The following brain injection treatments were administered: Ringer solution (130 mM of NaCl, 6 mM of KCl, 4 mM of MgCl_2_, 5 mM of CaCl_2_, 10 mM of HEPES, 25 mM of glucose, and 160 mM of sucrose) (control), 10 mg/mL of octopamine, and 3 mM of fumarate (a known octopamine synthesis inhibitor). After 10 min, to manipulate the hemolymph trehalose levels, the bees were injected with 1 µL in the thorax with one of the following treatments: Ringer’s solution (160 mM of NaCl, 6 mM of KCl, 8 mM of CaCl_2_, and 4 mM of MgCl_2_) (control) or 10% sorbose solution. A treatment of 10% sorbose was used to lower the trehalose levels of forager bees [12]. The harnessed bees underwent a proboscis extension response (PER) assay 5 min after the thorax injections to measure their appetite. This consisted of touching the bee’s antennae with the following ascending concentrations: 0%, 0.1%, 0.3%, 1%, 3%, 10%, and 30% of sucrose solution (*w*/*v*). In between concentrations, water was used to desensitize the bee’s antennae. We noted whether the bee extended its proboscis in attempt to feed on the sucrose solution. This is a standardized method for measuring appetite in the honey bee [42]. Immediately after the PER assay, the bees were flash frozen in liquid nitrogen and stored in the −80 °C freezer (Thermo Fisher Scientific, Waltham, MA, USA) until further analysis. The trials were carried out at the same time of day to account for circadian fluctuations in biogenic amine profiles. All chemicals were purchased from Sigma-Aldrich (Merck, Kenilworth, NJ, USA). For a summary of the experimental timeline, please see Figure 1.

### 2.3. HPLC Biogenic Amine Analysis

The analysis of the biogenic amines methods were based from [33]. Briefly, brains were dissected on dry ice and occipital lobes were removed using a scalpel and Zeiss stereo scope (Zeiss, Munich, Germany). A total of 3 brains were pooled together within a treatment group in 1.5 mL microcentrifuge tubes and these were placed on ice for 30 s. The brains were homogenized using a pestle in 50 µL of 0.2 M perchloric acid solution. The perchloric acid solution contained 100 pg/μL of N-methylserotonin oxalate and 50 pg/μL of synephrine, which served as internal standards. N-Methylserotonin oxalate was an indicator for dopamine and serotonin, while synephrine was used for octopamine and tyramine. Each sample was then sonicated for 5 min in ice water, and then placed on ice for another 20 min. The contents were centrifuged at 4 °C for 10 min with a speed of 15,000 g (Eppendorf centrifuge 5804 R, Hamburg, Germany). For HPLC analysis, the 50-µL supernatant was taken and transferred to a 2-mL dark amber microvial that contained a 100 µL of glass insert.

Samples were run on a Thermo Scientific Dionex Ultimate 3000 HPLC system consisting of a SR-300 solvent rack, a refrigerated ISO-3100BM pump, a WPS-3000TBSL analytical autosampler and an ECD-3000RS Electrochemical Detector (Thermo Fisher Scientific, Waltham, MA, USA). The autosampler was maintained at 5 °C, and a custom made 80 mm × 4.6 mm high-efficiency, C18 reverse-phase catecholamine, HR-80 column (3-μm particle packing) (Teknikus Kromatografi Teknolojileri, Istanbul, Turkey) was used and then maintained at 32 °C. Only 2 of the 4 channels of the ECD-3000RS electrochemical detector were used: one channel was set to 650 mV for tyramine and octopamine, while the other was set to 350 mV for serotonin and dopamine. The mobile phase consisted of 10% acetonitrile, 1.7 mM of 1-octanesulfonic acid sodium salt, 25 μM of EDTA tetrasodium tetrahydrate, and 75 mM of sodium dihydrogen phosphate monohydrate with a pH of 3, which was adjusted using concentrated phosphoric acid. The flow rate was set to 0.4 mL/min with an injection volume of 2 μL. The peaks were manually integrated based on the known standard retention times using the Chromeleon v. 7.2 software (Thermo Fisher Scientific, Waltham, MA, USA). External standards were run at the beginning and end of each batch of 48 samples. The samples were run in a randomized order. The biogenic amines were quantified on a per brain basis from standard curves. All chemicals were purchased from Sigma-Aldrich (Merck, Kenilworth, NJ, USA).

### 2.4. Statistical Analyses

All statistical analyses were conducted in JMP Pro v. 15 (SAS, Cary, NC, USA). The PER scores were transformed into a gustatory response score (GRS) by summing the responses across the sucrose solutions [44]. This was treated as count data and was analyzed using a generalized linear model with a Poisson distribution that corrected for overdispersion. This was followed by chi-square post hoc tests for multiple comparisons. Age and treatment of the brain and thorax were considered to be independent variables, while GRS was the dependent variable. To confirm successful biogenic amine manipulation, the biogenic amines were found to be not normally distributed, so they were analyzed using a Kruskal–Wallis test. These were conducted across the brain injection treatments within each age class and thorax injection treatment. A Bonferroni correction was used for the Wilcoxon post hoc multiple comparisons.

## 3. Results

### 3.1. Appetite Regulation

**Figure 2:**
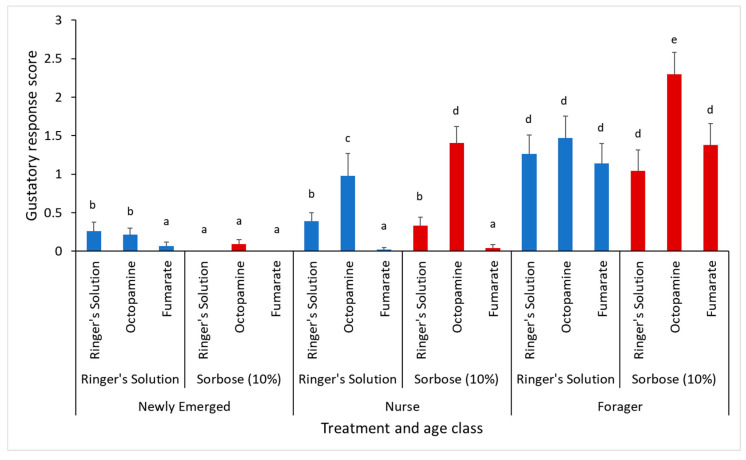
The gustatory response scores (GRS) measuring the appetite of the treated bees across the three age classes (newly emerged, nurse, and foragers). The control bees of each age class are indicated with blue bars and the bees treated with 10% sorbose are indicated in red. Each bar represents the mean GRS, while the error bars represented the standard error. The letters above each bar represent significant differences at the alpha = 0.05 level.

Overall, there was a significant difference across age classes with an increase in appetite as the bee ages (Generalized Linear Model: χ^2^_2,783_ = 174.97, *p* < 0.0001). The brain injections had a significant effect as well (χ^2^_2,783_ = 40.30388, *p* < 0.0001), but the thorax injections did not have a significant effect overall (χ^2^_1,783_ = 1.25436, *p* = 0.26). Fumarate significantly lowered appetite levels in the newly emerged bees injected with Ringer’s solution in the thorax and in both treatments of the nurse bees. However, there was no significant effect in the forager bees. Interestingly, within the nurse and forager age class, lowered trehalose levels from the 10% sorbose injections caused even higher appetite levels in bees injected with octopamine in the brain. An octopamine injection on its own only caused elevated appetite levels in the nurse age class (Figure 2).

### 3.2. Biogenic Amine Profiles

**Figure 3:**
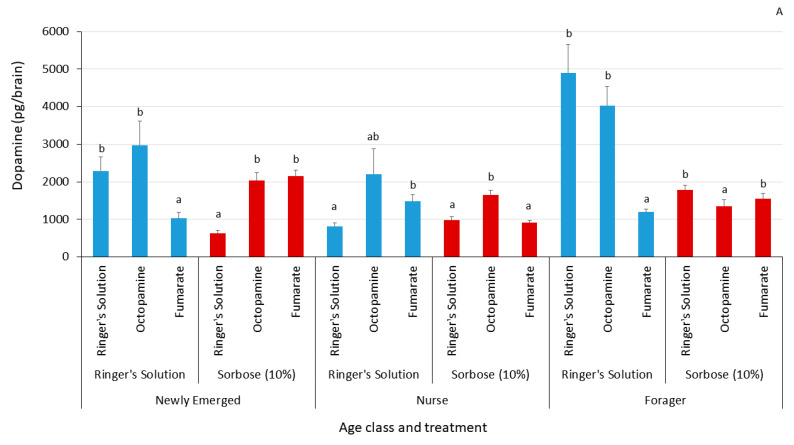

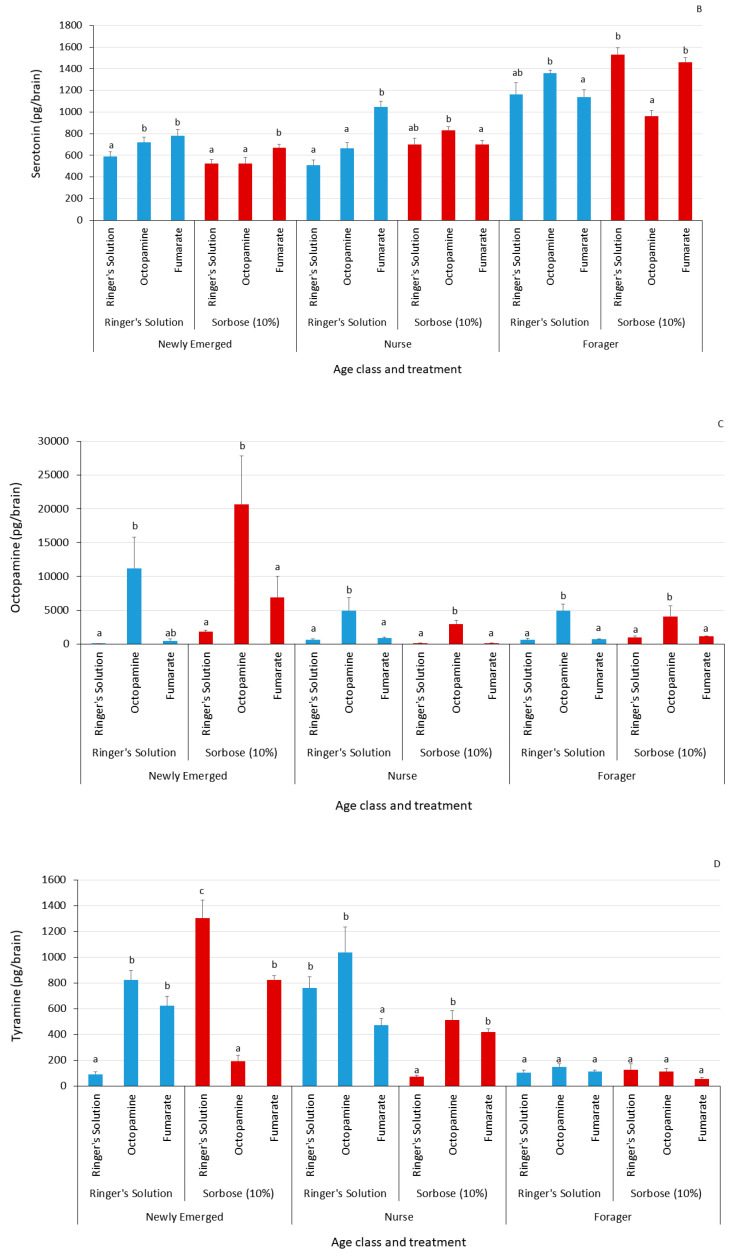
The biogenic amine levels for (**A**) dopamine, (**B**) serotonin, (**C**) octopamine, and (**D**) tyramine measured after the proboscis extension response assay at the end of the experiment. Each bar represents the mean and the error bars represent standard errors. The bars are grouped by the thorax injection treatment within their respective age class. Each bar corresponds to the brain injection treatment administered before the proboscis extension response assay. The letters above each bar represent significant differences at the alpha = 0.05 level.

The change in dopamine levels was both age- and treatment-dependent. Newly emerged bees experienced an increase in dopamine from octopamine and fumarate treatments with the 10% sorbose treatment, while fumarate caused a lowering in dopamine in the Ringer’s control group. In nurse bees, there was a lowering in dopamine levels from fumarate relative to the octopamine treated bees within the control group. However, in the 10% sorbose treated group there was an increase only in the octopamine treated bees. The forager bees had lower dopamine levels after fumarate treatment within the control group, and there was also a lowering in dopamine after octopamine and a 10% sorbose treatment (Figure 3A). Fumarate tended to increase serotonin levels in the newly emerged and nurse bees, except for the 10% sorbose-treated bees, while octopamine treatment lowered serotonin levels, but only in the forager bees that were treated with 10% sorbose (Figure 3B). The octopamine injections were successful at elevating octopamine baseline levels across all age classes relative to both the Ringer’s control and the fumarate treated bees (Figure 3C). In newly emerged bees, octopamine injections caused an increase in tyramine for the control group, but a decrease in the 10% sorbose treated group. Fumarate also caused an increase in tyramine levels within the control group and a decrease in the 10% sorbose treated group within the newly emerged bees. In nurse bees, fumarate caused a decrease in tyramine levels within the control group and an increase in the bees treated with 10% sorbose. Octopamine also caused an increase in tyramine levels in the 10% sorbose treated group of nurse bees. Foragers, on the other hand, had no fluctuation in tyramine levels due to any of the treatments administered (Figure 3D). A summary of the statistics can be found in Table 1.

## 4. Discussion

Within the nurse and forager age class, there appears to be an interactive effect between the lowering in hemolymph trehalose and higher baseline brain octopamine levels, which resulted in increased appetite levels. Furthermore, the effect was rather large because when nurse bees had lowered trehalose levels in the hemolymph and increased octopamine levels in the brain, they had the same appetite levels as the forager bees. Octopamine on its own did not increase appetite levels in forager bees and this was probably because the baseline octopamine appetite levels are already quite high in these bees and therefore there may be a ceiling effect that has been observed previously [18]. Octopamine increased appetite levels in forager bees with lowered trehalose levels. However, fumarate not lowering appetite levels in just the forager age class is an unexpected result. One possible explanation is that forager bees may have multiple pathways involved in appetite regulation [33], unlike the nurse bees, where fumarate consistently suppressed appetite levels as expected [45]. Another explanation for the lack of fumarate effect on forager bees could be that the octopamine was at such high concentrations, blocking the synthesis of more, in such a short time frame of 15 min, was not practical. Although it is important to note that, in the fumarate-treated nurse bees, there were no significantly lower octopamine levels in the brain, there was instead a fluctuation of the other three biogenic amines relative to the control. As previously shown, newly emerged bees have low-baseline appetite levels and do not seem to be very responsive to appetite regulatory mechanisms, such as higher octopamine levels or starvation [25,33].

We were able to successfully increase the octopamine baseline levels across all age classes to reveal its effects as the bee ages in relation to appetite regulation and this was confirmed based on our HPLC analysis. However, it is also important to note that, from both fumarate and octopamine injections, there were significant changes across the age classes and sugar manipulation treatments, for the rest of the biogenic amines as well. The effects of the brain injection treatments were not that consistent across the age classes and thorax injection treatments, so their effects on appetite regulation was not as clear. The unexpected effects from octopamine and fumarate injections highlights that it is best to account for all biogenic amines after pharmacological brain injections as they can cause unintended changes in the other biogenic amines besides the one of interest. The production of dopamine, tyramine, and octopamine are correlated with one another as they are derived from tyrosine and are from the same metabolic pathway [30], so changes in one is likely to affect the other during experimental manipulations. We cannot rule out the effects of dopamine and tyramine on appetite regulation because they are also known to influence appetite levels in honey bees [25]. Therefore, further research is required to elucidate how biogenic amines might interact with one another in order to regulate appetite levels in adult honey bees.

Yet, collectively, our results suggest that octopamine plays a role in appetite regulation within the nurse and forager age class and that there are age-dependent differences in appetite regulatory mechanisms in adult honey bees. We suspect that the naturally higher octopamine baseline levels in the forager bees results in this age class being more sensitive to the fluctuating trehalose levels in the hemolymph, which in turn results in more precise and rapid appetite regulation in this age class. Interestingly, fruit flies have a subset of octopaminergic neurons that initiates feeding behavior identified from knockdowns; however, when octopamine is administered, only the fed individuals showed a significant increase in appetite [46]. Furthermore, octopamine administered to fed and starved forager bees showed no significant differences in appetite levels [18]. These findings suggests that the effect of octopamine on appetite is also affected by the energetic state of the individual.

Newly emerged bees have already been shown to increase their appetitive learning levels from higher octopamine levels in the brain [16], but here we show that this does not increase their appetite levels. This finding is supported by previous work where higher octopamine levels in starved forager bees, but not nurse and newly emerged bees, were correlated with higher appetite levels [33]. This unique appetite regulatory mechanism in forager bees is possibly more efficient than relying on the glucose–insulin signaling pathway in which hemolymph glucose levels are monitored instead. Supporting this notion, sugar levels were not affected from ILP1 and ILP2 knockdowns in bee larvae, but both appetite and sugar levels are affected by Vg and JH knockdowns, which are linked to octopamine production [47,48].

Honey bees store and build up trehalose in their hemolymph rather than glucose, so trehalose is thought to be the best indicator of the individual’s energetic state [3,6,12,13]. Consequently, using trehalose levels to regulate appetite may be more efficient for forager bees, and there may be a higher selection pressure on them for this. Forager bees are carrying out energetically expensive activities, such as sustaining flight when foraging [49], but yet do not have any available food sources on hand as if they were in the hive. The octopamine levels that naturally increase as the bee ages could therefore be a possible mechanism by which forager bees are more in tune with their energetic state at any given moment. This appetite regulatory mechanism would likely help safe guard them against starving to death while foraging outside the hive.

## Figures and Tables

**Table 1:** A summary of the statistical output from the Kruskal–Wallis tests carried out on the biogenic amine levels within each age class and thorax injection treatment, across each brain injection treatment. The * besides the *p*-value indicates significance at the alpha = 0.05 level. The change in the biogenic amine levels relative to the control is indicated within the last column, a + indicates significantly higher level, while–indicates significantly lower levels. None indicate no significant changes in the biogenic amine level relative to the control.

Biogenic Amine	Age Class	Thorax Injection	Brain Injection	Sample Size	Chi Square Value	*p*-Value	Change Relative to Control
Dopamine	Newly emerged	Ringer’s solution	Ringer’s solution	11	15.1079	0.0005 *	
			Octopamine	14			None
			Fumarate	10			-
		Sorbose (10%)	Ringer’s solution	16	29.5316	<0.0001 *	
			Octopamine	12			+
			Fumarate	15			+
	Nurse	Ringer’s solution	Ringer’s solution	14	8.2859	0.0159 *	
			Octopamine	13			None
			Fumarate	14			None
		Sorbose (10%)	Ringer’s solution	15	23.4280	<0.0001 *	
			Octopamine	16			+
			Fumarate	16			None
	Forager	Ringer’s solution	Ringer’s solution	16	21.3882	<0.0001 *	
			Octopamine	15			None
			Fumarate	17			-
		Sorbose (10%)	Ringer’s solution	17	11.4352	0.0033 *	
			Octopamine	17			-
			Fumarate	17			None
Serotonin	Newly emerged	Ringer’s solution	Ringer’s solution	11	7.3363	0.0255 *	
			Octopamine	14			None
			Fumarate	10			+
		Sorbose (10%)	Ringer’s solution	18	5.0325	0.0808	
			Octopamine	12			None
			Fumarate	15			None
	Nurse	Ringer’s solution	Ringer’s solution	14	24.2077	<0.0001 *	
			Octopamine	13			None
			Fumarate	14			+
		Sorbose (10%)	Ringer’s solution	15	6.0672	0.0481 *	
			Octopamine	16			None
			Fumarate	16			None
	Forager	Ringer’s solution	Ringer’s solution	16	4.0421	0.1325	
			Octopamine	15			None
			Fumarate	17			None
		Sorbose (10%)	Ringer’s solution	17	27.3048	<0.0001 *	
			Octopamine	17			-
			Fumarate	17			None
Octopamine	Newly emerged	Ringer’s solution	Ringer’s solution	11	24.2461	<0.0001 *	
			Octopamine	14			+
			Fumarate	10			None
		Sorbose (10%)	Ringer’s solution	18	9.2173	0.0100 *	
			Octopamine	12			+
			Fumarate	15			None
	Nurse	Ringer’s solution	Ringer’s solution	14	21.8426	<0.0001 *	
			Octopamine	13			+
			Fumarate	14			None
		Sorbose (10%)	Ringer’s solution	15	31.0247	<0.0001 *	
			Octopamine	16			+
			Fumarate	16			None
	Forager	Ringer’s solution	Ringer’s solution	16	29.8394	<0.0001 *	
			Octopamine	15			+
			Fumarate	17			None
		Sorbose (10%)	Ringer’s solution	17	5.7434	0.0566	
			Octopamine	17			+
			Fumarate	17			None
Tyramine	Newly emerged	Ringer’s solution	Ringer’s solution	11	23.2475	<0.0001 *	
			Octopamine	14			+
			Fumarate	10			-
		Sorbose (10%)	Ringer’s solution	18	27.9985	<0.0001 *	
			Octopamine	12			+
			Fumarate	15			-
	Nurse	Ringer’s solution	Ringer’s solution	14	8.5954	0.0136 *	
			Octopamine	13			None
			Fumarate	14			-
		Sorbose (10%)	Ringer’s solution	15	30.5213	<0.0001 *	
			Octopamine	16			+
			Fumarate	16			+
	Forager	Ringer’s solution	Ringer’s solution	16	4.3956	0.1110	
			Octopamine	15			None
			Fumarate	17			None
		Sorbose (10%)	Ringer’s solution	17	2.4679	0.2911	
			Octopamine	17			None
			Fumarate	17			None

## Data Availability

All raw data has been made publicly available and are stored within the following link https://osf.io/wukn2/, accessed on 20 September 2021.
